# Mannose-Binding Lectin 2 Polymorphisms Do Not Influence Frequency or Type of Infection in Adults with Chemotherapy Induced Neutropaenia

**DOI:** 10.1371/journal.pone.0030819

**Published:** 2012-02-17

**Authors:** Michelle Wong, Lars Öhrmalm, Kristina Broliden, Carl Aust, Martin Hibberd, Thomas Tolfvenstam

**Affiliations:** 1 Infectious Disease Unit, Department of Medicine, Karolinska University Hospital, Karolinska Institutet, Stockholm, Sweden; 2 Genome Institute of Singapore, Singapore, Singapore; University of Bergen, Norway

## Abstract

**Background:**

Mannose-binding Lectin protein (MBL) has been suggested to be relevant in the defence against infections in immunosuppressed individuals. In a Swedish adult cohort immunosuppressed from both the underlying disease and from iatrogenic treatments for their underlying disease we investigated the role of MBL in susceptibility to infection.

**Methods:**

In this cross sectional, prospective study, blood samples obtained from 96 neutropaenic febrile episodes, representing 82 individuals were analysed for single nucleotide polymorphism (SNP) in the *MBL2* gene. Concurrent measurement of plasma MBL protein concentrations was also performed for observation of acute response during febrile episodes.

**Findings:**

No association was observed between *MBL2* genotype or plasma MBL concentrations, and the type or frequency of infection. Adding to the literature, we found no evidence that viral infections or co-infections with virus and bacteria would be predisposed by MBL deficiency. We further saw no correlation between *MBL2* genotype and the risk of fever. However, fever duration in febrile neutropaenic episodes was negatively associated with *MBL2* SNP mutations (*p*<0.05). Patients with *MBL2* SNP mutations presented a median febrile duration of 1.8 days compared with 3 days amongst patients with wildtype *MBL2* genotype.

**Interpretation:**

We found no clear association between infection, or infection type to *MBL2* genotypes or plasma MBL concentration, and add to the reports casting doubts on the benefit of recombinant MBL replacement therapy use during iatrogenic neutropaenia.

## Introduction

Mannose-Binding Lectin protein (MBL) is a member of the collectin family of proteins which function as pattern recognition molecules in the innate immune system. MBL recognises and binds to sugar groups present on a wide range of microorganism and plays a significant role as a first line defence against invading pathogens by triggering the complement pathway using MBL-associated serine proteases and possibly functioning as a toll-like receptor co-receptor [1], [2]. Single nucleotide polymorphisms (SNPs) located within the promoter region and exon 1 of *MBL2* have been correlated with MBL serum levels [3]. Within exon 1, the wildtype variant is termed ‘A’ and relevant SNP mutations at positions +154 (D variant), +161 (B variant) and +170 (C variant) are collectively denoted by ‘O’. In the promoter regions, three relevant SNP loci have been identified at positions -619 (H/L), −290 (X/Y) and −66(P/Q). In the presence of wildtype ‘A’, HYP, LYP and LXP have been associated with high, intermediate and low MBL levels respectively [3], [4]. The combinatorial effect of SNPs in the promoter and coding region of *MBL2* results in variations in MBL concentrations [3], [4]. Whilst constitutional MBL deficiency is unlikely to be clinically relevant in healthy adults [5], this may not be the case in patients with myelosuppression from chemotherapy. Antineoplastic chemotherapy primarily affects the cellular components of the adaptive and innate immune system rendering acellular components of the innate immune system such as MBL potentially important in the role of microbial defense.

Infection episodes are common in patients after induction of chemotherapy during the subsequent neutropaenic phase and account for significant mortality [6]. In 2001, Neth et al. reported a significant increase in the duration and frequency of chemotherapy-induced neutropaenic febrile episodes amongst children with serum MBL deficiency [7]. Subsequent studies in adult patients treated with antineoplastic chemotherapy have yielded conflicting conclusions and metanalyses of data from adult patients are lacking. Low plasma protein levels of MBL has been associated to serious infections related to chemotherapy by Peterslund et al., and Vekemans et al. conclude that while MBL deficiency does not predispose to more frequent or prolonged febrile episodes during myelosuppressive therapy, an association with more severe infections exist [8], [9]. In contrast, Bergmann et al. and Kilpatrick et al. dismiss any strong relationship between low MBL levels and febrile neutropaenia [10], [11]. Furthermore, Lopez et al. and Klostergaard et al. did not find an association between *MBL2* genotype and number of infectious episodes following chemotherapy against follicular lymphoma and occurrence of septicaemia, respectively [12], [13].

Comparisons between previous studies are inherently complicated by different distribution of haematological malignancies in the cohorts, different therapies administered and measurement of either serum MBL levels or *MBL2* genotype and induction of MBL by inflammation could introduce a temporal sampling bias in studies relying on only protein measurement. Furthermore, association to infection aetiology, apart from unspecified bacteraemia, has yet not been performed. In addition to earlier reports on MBL binding to viral glycoproteins [14], [15], MBL has recently been shown to have protective effects against Ebola virus in an *in vivo* murine model [16].

As recombinant human MBL has been put through phase I studies [17] based on the suggestion that it is able to decrease infection-related complications in MBL deficient patients with iatrogenic neutropaenia, further study of the role of MBL and other innate constituents in granulocytopaenia are warranted. Here, we sought to study the association of plasma MBL levels and *MBL2* coding and promoter region genotypes to infection, type of infection and, febrile episodes in adults with chemotherapy induced neutropaenia.

## Materials and Methods

### Study population

The study was approved by The Regional Ethical Review Board in Stockholm, permit numbers 2007/1213-31/4 and 2008/1300-32.

During a 26 month period (January 2008 to February 2010), adult patients with haematological disorders at the Karolinska University Hospital, Stockholm were, after informed written consent, included in a cross sectional study where the inclusion criterion was chemotherapy-induced neutropaenia (absolute neutrophile count ≤500/mm^3^). Patients that developed fever (auricular temperature >38·0°C twice within an hour or ≥38·5°C at one occasion) were sampled within 72 hours from fever onset, whereas patients without fever were sampled upon routine medical appointments during the neutropaenia episode. Whole blood was collected in EDTA-tubes; plasma and blood clots were stored separately at −80°C until use for MBL measurement and genomic DNA extraction, respectively. Additional blood and nasal pharyngeal aspirates (NPA) samples were collected for microbiological testing. C-reactive protein (CRP) concentrations were measured as a clinical routine on the sampling day and acquired from the medical records. Medical records were retrospectively acquired for all included patients. Data was collected from diagnosis of the haematological disorder, through all neutropaenic episodes and ceased upon the patient's recovery, demise or study closure (June 2010), whichever was earlier. Episodes associated with patients migrating into the Stockholm area after initiation of treatment had been excluded from the retrospective, patient-centric analysis but still included in episode-centric analysis pertaining to *MBL2* genotypes and the corresponding plasma protein levels. Patients that underwent liver transplants were excluded since MBL is produced in the liver and genotypes may vary between the donor and recipient. In the same manner, patients that had undergone allogeneic haematopoietic stem cell transplantation (HSCT) were also excluded.

### *MBL2* genotyping

Six SNPs, rs11003125 (L/H allele), rs7096206 (X/Y allele), rs7095891 (P/Q allele), rs5030737 (D variant), rs1800450 (B variant) and rs1800451 (C variant) were investigated. DNA was extracted from the blood clots with QIAamp blood DNA midi kit (Qiagen) according to the manufacturer's protocol and SNP alleles were subsequently determined by sequencing. A nested PCR protocol was designed to selectively amplify the promoter and exon 1 regions of the *MBL2* gene. A pair of primers, *MBL2*_Out_F -5′- TTGCCAGTGGTTTTTGACTC-3′ and *MBL2*_Out_R-5′-TGCCAGAGAATGAGAGCTGA -3′ was first used to amplify a 1079 bp segment of the *MBL2* gene, including the six SNP loci. PCR amplification was performed in a 50 µl reaction consisting 5 µl DNA template, 1× *pfu* PCR buffer, 200 µM dNTP, 400 nM of each primer and 1·5 U *pfu* DNA polymerase (Promega Corporation). Amplification was carried out with an initial denaturation at 95°C for 2 minutes, followed by 35 cycles of 95°C for 1 minute, 65°C for 1 minute and 72°C for 1 minute, and a final extension at 72°C for 7 minutes. PCR products from the above reaction were then used for further amplification prior to sequencing. Two pairs of primers were designed to flank the SNPs in exon 1 and the promoter regions respectively. Primers for the promoter region are *MBL2*_PROMOTER_F-5′-TTCCTGCCAGAAAGTAGAGAGG-3′ and *MBL2*_PROMOTER_R-5′- GGATCCTAAGGAGGGGTTCA-3′ whilst primers for the exon 1 region are *MBL2*_EXON1_F-5′- AGTCACGCAGTGTCACAAGG-3′ and *MBL2*_EXON1_R-5′- CAGGCAGTTTCCTCTGGAAG-3′. PCR amplification mix for the promoter region consists of 1 µl PCR product from the Out primer, 1× *pfu* PCR buffer, 200 µM dNTP, 400 nM of each primer, 1·5 U *pfu* DNA polymerase and water to a final volume of 50 µl. PCR cycling condition begins with an initial denaturation at 95°C for 2 minutes, followed by 35 cycles of 95°C for 1 minute, 55°C for 1 minute and 72°C for 1 minute, and a final extension step at 72°C for 7 minutes. For the exon 1 region, the PCR amplification mix consists of 1 µl PCR product from the out primer, 1× *pfu* PCR buffer, 200 µM dNTP, 800 nM of each primer, 1.5 U *pfu* DNA polymerase and water to a final volume of 50 µl. PCR cycling condition begins with an initial denaturation at 95°C for 2 minutes, followed by 35 cycles of 95°C for 1 minute, 65°C for 1 minute and 72°C for 1 minute, and a final extension step at 72°C for 7 minutes. PCR products from promoter and exon 1 region were then purified with QIAquick gel extraction kit (Qiagen) after separation with 1.5% agarose and sequenced with ABI3730XL after ABI BigDye Terminator v3.1 (Applied Biosystems) reactions. SNP alleles were then determined according to the sequencing chromatograms.

### MBL plasma concentrations

MBL plasma concentrations were measured with a commercially available MBL oligomer ELISA kit (Bioporto Diagnostics). The dynamic range for the assay was 0.5 µg/L −40 µg/L and plasma samples were diluted 100 to 1000 times for measurement within this range.

### Microbiology

Bacteria were cultured from blood and PCR viral diagnostics for adenovirus, Epstein - Barr virus and cytomegalovirus were performed, as per normal clinical routine, by the local clinical microbiology laboratory, Karolinska University Hospital. Viral diagnostics for BK polyomavirus from blood samples and, respiratory viruses from NPA samples were however, based on PCR methods described in previous studies [18], [19], [20], [21], [22], [23], [24] and Ohrmalm et al [25].

### Statistical analysis

Statistical analyses were made with the Prism suite (Graphpad Inc). Fisher's exact test and Mann-Whitney were used for investigating the patients' general characteristics while the chi-squared test was used for determining congruence of SNP prevalence with a reference Danish population and determining Hardy-weinberg equilibrium. Kruskal-wallis test was used for all other analyses where comparison of continuous data for 3 groups or more were required.

## Results

**Figure 1:**
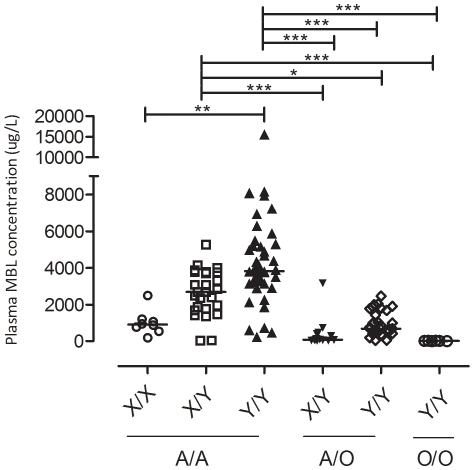
*MBL2* XA/XA, XA/YA, YA/YA, XA/YO, YA/YO, YO/YO genotypes with the corresponding plasma MBL concentrations (**p<0.01, ^***^ *p*<0.001).

A total of 109 episodes of fever during neutropaenia were sampled and 13 were excluded due to insufficient genomic material for genotyping. Thirty-three afebrile neutropaenic episodes were also included as controls. This amounted to 82 febrile neutropaenic patients and 26 afebrile neutropaenic patients (Table 1 and 2). The majority of patients were treated for acute leukaemias and none were diagnosed with invasive fungal infections. Genotype frequencies for the six SNPs investigated, and seven common haplotypes were in Hardy-weinberg equilibrium (Table 3) and were congruent with previous studies conducted amongst healthy Danes [3], [4]. MBL concentrations in our study were also similar to those observed in another chemotherapy-induced neutropaenic cohort [10]. In line with previous studies, mutations in the *MBL2* coding region corresponded to a reduction (*p*<0.001) of plasma MBL concentrations (Table 2). Amongst A/A individuals, mutations in the H/L SNP locus did not result in any significant change in MBL concentrations. X/Y locus mutations correlated with significantly (*p*<0·001) reduced MBL concentrations and the much less studied P/Q locus contributed to significant (*p* = 0·0149) concentration reduction as well. Hence, in the presence of functional MBL protein, the impact of the promoter and P/Q SNPs increase in the following order; H/L<P/Q<X/Y. With A/O individuals, the P/Q SNP did not have significant impact on MBL concentrations. Instead, mutations in the H/L and X/Y SNP loci were able to significantly (*p*<0·001 and *p* = 0·006, respectively) reduce MBL concentrations. The number of O/O individuals included in this study was too few for further analysis. Taking into account the correlation of X/Y allele in both the A/A and A/O individuals, additional classification into XA/XA, XA/YA, YA/YA, XA/YO, YA/YO and YO/YO was performed and the corresponding median MBL concentrations differed significantly (*p<0.0001)* (Fig. 1b).

**Table 1:** General characteristics of febrile and afebrile neutropaenic episodes.

	Fever				Without fever			
	*MBL2* coding regions				*MBL2* coding regions			
	A/A	A/O+O/O			A/A	A/O+O/O		
	(n = 55)	(n = 41)	*p*-value	OR (95%CI)	(n = 20)	(n = 13)	*p*-value	OR (95%CI)
Days to febrile episode, median (IQR)	2 (1.0–5.25)	3 (1.0–4.25)	0.94	-	-	-	-	-
Underlying disease								
Acute Leukaemia (%)	31 (57.4)	23 (50.0)	0.55	1.35 (0.61–2.97)	10 (47.6)	3 (20)	0.159	3.64 (0.789–16.76)
Chronic Lymphocytic Leukaemia (CLL) (%)	1 (1.9)	2 (4.3)			0	2 (13.3)	-	-
Hodgkin Lymphoma (%)	1 (1.9)	2 (4.3)			3 (14.3)	2 (13.3)	-	-
Myeloma (%)	8 (14.8)	5 (10.9)	0.77	1.43 (0.43–4.71)	0	1 (6.7)	-	-
Non-Hodgkin's lymphoma (NHL)(%)	13 (24.1)	13 (28.3)	0.65	0.80 (0.33–1.97)	8 (30.1)	6 (40.0)	1.00	0.92 (0.237–3.589)
Other haematological malignancy (%)	0 (0.0)	1 (2.2)			0	1 (6.7)	-	-
Treatment type								
Antineoplastic chemotherapy (%)	52 (96.3)	42 (91.3)	1.00	1.24 (0.24–6.46)	19 (90.5)	14 (93.3)	1.00	0.68 (0.06–8.25)
Steroids (%)	9 (16.7)	13 (28.3)	0.15	0.48 (0.18–1.26)	3 (14.3)	2 (13.3)	1.00	1.08 (0.16–7.44)
Monoclonal antibodies (%)	7 (13.0)	5 (10.9)	1.00	1.17 (0.34–3.96)	2 (9.5)	1 (6.6)	1.00	1.47 (0.12–17.92)
Cyclosporine A or takrolimus (%)	0 (0.0)	1 (2.2)			1 (4.8)	0 (0.0)	1.00	2.27 (0.09–59.6)

**Table 2:** MBL concentrations and microbiological findings of febrile and afebrile neutropaenic episodes.

	Fever					Without fever						
	*MBL2*coding regions					*MBL2* coding regions						
	A/A	A/O+O/O	Total			A/A	A/O+O/O	Total				
	(n = 55)	(n = 41)		*p*-value	OR (95%CI)	(n = 20)	(n = 13)		*p*-value	OR (95%CI)	*p-value* 1	*p-value* 2
MBL concentrations, µg/L (IQR)	3372 (2089.0–4187.0)	645.7(80.3–1314.0)	-	<0.001	-	3508 (2184–4659)	431.8(185.6–606.2)		<0.002	-	0.6839	0.2952
Days to febrile episode, median (IQR)	2 (1.0–5.25)	3 (1.0–4.25)	-	0.94	-	-	-	-	-	-	-	-
No. with bacteraemia (%)	23 (42.6)	12 (26.0)	35	0.09	2.10 (0.90–4.92)	N.D	N.D.	N.D.	-	-	-	-
No. with viral finding in blood or NPA (%)	24(44.4)	15(32.7)	39	0.31	1.55 (0.64–3.51)	2(9.5)	2 (13.3)	4	-	-	-	-
No. with no detectable pathogen (%)	18 (33.3)	19 (35.2)	37	0.53	0.71 (0.31–1.61)	19 (90.5)	13 (86.7)	32	1.000	1.46 (0.182–11.74)	-	-
No. with severe neutropaenia, <0.1×10^9^cells/L (%)	36 (66.7)	31 (67.4)	73	1.00	1.07 (0.46–2.50)	8 (38.1)	7 (46.7)	15	0.74	0.70 (0.18–2.70)	-	-

1 Comparison of MBL concentrations from episodes of A/A individuals with fever and without fever.

2 Comparison of MBL concentrations from episodes of A/O and O/O individuals with fever and without fever.

N.D. Bacteria investigations were not performed as a routine.

**Table 3:** *MBL2* genotype frequencies observed in this cohort (n = 108).

Genotype			Haplotypes	
Promoter				n (%)
dbSNP ID	allele	n (%)	HYPA	64 (29.6)
rs11003125	L/L	47 (43.5)	LYPA	13 (6.0)
	L/H	46 (42.6)	LYQA	43 (19.9)
	H/H	15 (13.9)	LXPA	45 (20.8)
			LYPB	35 (16.2)
rs7096206	Y/Y	67 (62.0)	LYQC	4 (1.9)
	Y/X	34 (31.5)	HYPD	12 (5.6)
	X/X	7 (6.5)	LYPD	0 (0.0)
rs7095891	P/P	69 (63.9)		
	P/Q	31 (28.7)		
	Q/Q	8 (7.4)		

Exon 1				
dbSNP ID	allele	n (%)		
rs5030737	A/A	96 (88.9)		
	A/D	12 (11.1)		
	D/D	0 (0.0)		
rs1800450	A/A	75 (69.4)		
	A/B	30 (27.8)		
	BB	3 (2.8)		
rs1800451	A/A	104 (96.3)		
	A/C	4 (3.7)		
	C/C	0 (0.0)		
Total	A/A	63 (58.3)		
	A/O	39 (36.1)		
	O/O	6 (5.6)		

As the promoter SNP, X/Y played a secondary role in relation to the coding region SNPs in correspondence to MBL concentrations all further analyses were limited to mutations in the coding region and the X/Y locus only. All blood samples were collected within 72 hours of fever onset and time-dependent differences in MBL concentrations was not observed within this time-frame (*p* = 0·747). Underlying diseases, treatment regime and absolute neutrophile counts, were investigated and shown not to be confounding factors to the MBL concentrations (Table 1, 2).

**Figure 2:**
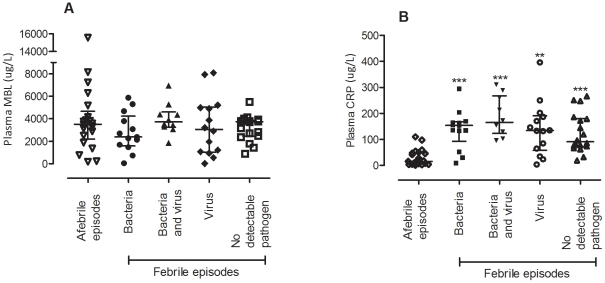
Plasma concentrations of (A) MBL and (B) CRP, in afebrile and febrile episodes based on pathogen detected. Comparisons were made between afebrile episodes and febrile episodes with further catergorisation based on microbiological findings. Numbers in the respective groups are listed in Table 2 (**p<0.01, ***p<0.001).

Based on genotype, individuals were classified into 2 groups, wildtype (A/A) and mutants (A/O, O/O). Mutations in the *MBL2* coding region could result in an unstable protein oligomer consequently, episodes arising from A/A individuals were used for analysis on microbiological findings. In an episode-centric analysis, MBL protein concentrations from A/A individuals with febrile episodes did not vary with bacterial or viral findings, co-occurrence of bacteria and virus or the absence of microbiological findings (Fig. 2a). Furthermore, no association was observed between MBL concentration and fever presentation, as shown by comparison between febrile and afebrile neutropaenic episodes. For comparison, CRP concentrations differed significantly between neutropaenic patients with and without fever (p<0·001) (Fig. 2b). Amongst episodes with virus findings, no significant differences in MBL concentrations were observed between virus positive samples taken from blood and nasopharynx (Table 4). In blood, the most common virus finding was BK polyomavirus and cytomegalovirus while in the NPA samples, the most common finding was human rhinovirus. Corresponding analyses with XA/XA, XA/YA, YA/YA, XA/YO, YA/YO and YO/YO genotypes did not yield any correlation either.

**Table 4:** Plasma MBL concentrations in wildtype (A/A) individuals with virus findings.

	Location of virus findings		
	Blood	Nasopharynx	*p*-value
MBL concentrations (µg/L) (IQR)			
Febrile patients	n = 16	n = 8	
	3487(2945–4756)	3167(1172–6448)	0.9756
Afebrile controls	n = 1	n = 1	
	6287	771.3	*-*

**Figure 3:**
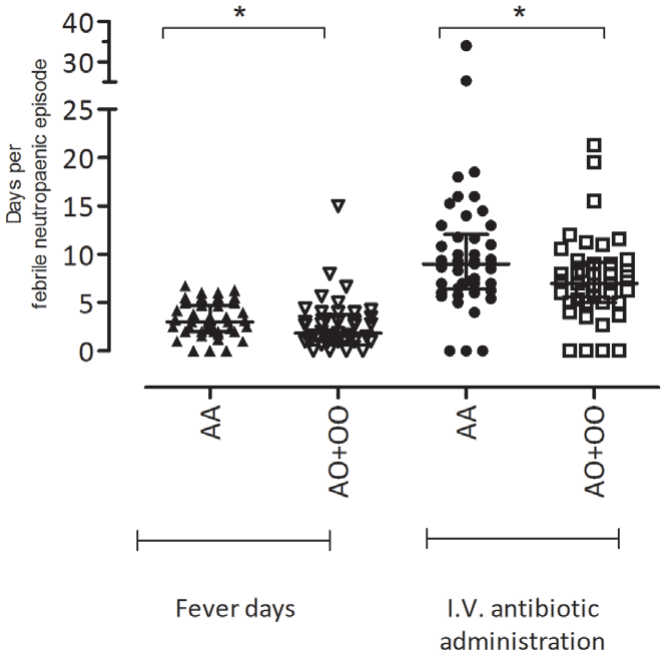
The proportional days of fever and days of antibiotic treatment during febrile neutropaenia episodes among A/A (n = 46) and A/O+O/O (n = 41) individuals. (^*^ ***p*** **<0.05).**

Patient-centric analysis was used for investigations on clinical outcome. No correlation could be observed between *MBL2* genotype and the risk of fever. However, the proportional days of fever during febrile neutropaenic episodes was weakly negatively associated with the presence of *MBL2* SNP mutations (*p* = 0·041). Patients with *MBL2* SNP mutations (A/O an O/O) presented a median febrile duration of 1·8 days (IQR = 1·0–3·8) compared with 3 days (IQR = 2·0–4·7) amongst patients with wildtype (A/A) *MBL2* genotype (Fig. 3). Antibiotic use corresponded closely to the manifestation of fever and the same trend was observed. Antibiotic treatment duration in wildtype A/A individuals were longer (*p* = 0·018) than in individuals with *MBL2* SNP mutations (A/O and O/O), with a median of 9·0 and 7·0 days, respectively. This association, was however, not observed with the when the X/Y locus was included in the analysis (XA/XA, XA/YA, YA/YA, XA/YO, YA/YO, YO/YO) (data not shown).

## Discussion

Our study set out to investigate, in an episode-centric analysis, the role of MBL as a risk factor for infection and type of infection among adults presenting with neutropaenic febrile episodes after undergoing chemotherapy for their underlying haematological malignancies. We further utilised patient-centric analysis to investigate associations between *MBL2* genotype and risk of fever in this cohort.

In the absence of Swedish genotype frequencies for the *MBL2* gene, Danes were selected as a reference population for our study. Whilst there have been other SNP loci identified for *MBL2* [26], [27], [28], our investigations focussed on 6 SNPS that had been previously reported to have an effect on protein concentrations [3]. Nonetheless, we had looked at additional SNPs [27] and saw that our cohort was homozygous for all the SNP loci except for the rs10556764 deletion, rs34120190, rs11003124, rs7084554, rs36014597 and rs10556764 where the frequency for the minor allele was low and could not be included for statistical analysis. The genotype frequencies of the *MBL2* SNPs we examined corresponded well with previous investigations of a Danish cohort, however, median MBL concentrations observed in both the wildtype (A/A) and mutant (A/O, O/O) individuals in our cohort were seemingly higher than that reported on healthy Danes [3], [4] suggesting that MBL concentrations are already elevated at the time of sampling, regardless of any ensuing infection. This finding could derive from inflammation caused by the malignant disease itself and has been observed in a similar cohort [10]. Indeed, MBL has been shown to bind directly to transformed cell lines exhibiting an aberrant glycosylation pattern [29]. The preceding chemotherapy cycle could also have contributed via MBL binding to apoptotic cells and clearance of dying cells through phagocytosis [30]. Although MBL concentrations were found to be higher than in healthy individuals, concentrations were not significantly different between neutropaenic patients with fever and without fever. Furthermore, MBL concentrations between patient groups diagnosed with bacterial, viral or, bacterial and viral co-infections did not differ significantly. In-depth analysis of MBL concentrations and the location of virus findings did not offer more clarity on the potential antiviral role of MBL in this patient cohort. This said it is still possible that any concurrent increase in response by MBL to infection could be obscured by elevation elicited by the neoplasm, or massive cell-death induced by cytotoxic therapy. Little is known regarding the kinetics of MBL secretion but Neth et al. suggested that a peak was seen only seven days after the start of a febrile neutropaenic episode [7]. We collected all samples within 72 hours from the start of a febrile neutropaenic episode which might have been premature for any observation of MBL infection kinetics between pathogen types. Nevertheless, we could not find any association between MBL concentration and time between fever onset and sampling. With another acute phase protein, CRP, differences were apparent between patients that developed fever during neutropaenia and, those that did not. Amongst the group with fever, no difference in CRP was observed regardless of pathogen type, in concurrence with MBL concentrations. The latter observation is not surprising since these patients are, as an effect of their cytopaenia, limited in their ability for IL-1 and IL-6 response. A negative correlation between CRP, IL-1 and MBL has been observed by Aittoniemi et al. among febrile septic patients indicating that MBL and CRP are regulated differently [31]. Arai et al. further demonstrated in haepatoma cell lines that IL-6 stimulates MBL production while IL-1 inhibits it [32]. Taken together, MBL elevation by infection is unlikely to impact the association between MBL concentrations and predisposition to infection.

In our hands, no association was observed between *MBL2* genotype or MBL concentrations and the type of infection or the frequency of infection in chemotherapy induced neutropaenia in adults. This is in stark contrast to a study performed by Peterslund et al. in a similar adult cohort [8]. Vekemans et al., conclude that MBL deficiency is associated to severe infection episodes defined as pneumonia, septicaemia or invasive fungal infection [9]. It could be argued that our material could lack sufficient statistical power to detect an association to severe infection, which, in our cohort is represented by clinically significant bacteraemia (*p* = 0·09). However, Klosterggard et al. focused only on this association and found no *MBL2* genotype associations to sepsis or death by sepsis [13]. In line with our data are several reports where no strong association between infection and MBL deficiency were found, but meta-analyses are missing [10], [11], [12]. Adding to the literature, we found no evidence that viral infections or co-infections with virus and bacteria would be predisposed by MBL deficiency. A weak association in our material was that mutations in the *MBL2* exon 1 region recorded fewer febrile days per febrile neutropaenic episode (*p* = 0·04), in covariance with time of antibiotic treatment. While this observation may be elicited by chance itself it contradicts the findings of Neth et al. who suggested that *MBL2* mutations predisposed longer febrile duration among children undergoing chemotherapy [7]. Interestingly, no association of the same clinical parameters and the promoter SNP X/Y, which had profound effect on MBL concentration, was apparent. An earlier report on the role of *MBL2* polymorphisms and recurrent respiratory infections in children suggested that it was the coexistence of (partial) immune defects, rather than any one single immunological aetiologic factor that was associated to the disease outcome [33]. We could speculate that this might contribute to the weak associations we observe and suggest consideration of this notion, originally suggested by Turner MW [34], for future studies [35].

In summary, in this adult cohort we found no clear association between infection, or infection- type to *MBL2* genotypes or MBL concentration, and add to the reports casting doubts on the benefit of recombinant MBL replacement therapy use during iatrogenic neutropaenia.
